# NMR quantification of diffusional exchange in cell suspensions with relaxation rate differences between intra and extracellular compartments

**DOI:** 10.1371/journal.pone.0177273

**Published:** 2017-05-11

**Authors:** Stefanie Eriksson, Karin Elbing, Olle Söderman, Karin Lindkvist-Petersson, Daniel Topgaard, Samo Lasič

**Affiliations:** 1 Division of Physical Chemistry, Department of Chemistry, Lund University, Lund, Sweden; 2 Department of Experimental Medical Science, Lund University, Lund, Sweden; 3 CR Development AB, Lund, Sweden; National Research Council of Italy, ITALY

## Abstract

Water transport across cell membranes can be measured non-invasively with diffusion NMR. We present a method to quantify the intracellular lifetime of water in cell suspensions with short transverse relaxation times, *T*_2_, and also circumvent the confounding effect of different *T*_2_ values in the intra- and extracellular compartments. Filter exchange spectroscopy (FEXSY) is specifically sensitive to exchange between compartments with different apparent diffusivities. Our investigation shows that FEXSY could yield significantly biased results if differences in *T*_2_ are not accounted for. To mitigate this problem, we propose combining FEXSY with diffusion-relaxation correlation experiment, which can quantify differences in *T*_2_ values in compartments with different diffusivities. Our analysis uses a joint constrained fitting of the two datasets and considers the effects of diffusion, relaxation and exchange in both experiments. The method is demonstrated on yeast cells with and without human aquaporins.

## Introduction

Specialized proteins known as aquaporins are key to transport of water across biological membranes [1]. Interesting historical perspectives on the discovery of aquaporins can be found in [2] and [3]. Nuclear magnetic resonance (NMR) has played an important role in quantification and understanding of water exchange across cell membranes. A system that has been extensively studied by NMR are red blood cells [4, 5]. To measure water exchange in cell suspensions, NMR experiments require a contrast between intracellular and extracellular water compartments. Most commonly, this contrast is provided by differences in relaxation rates [6] or apparent diffusion coefficients (ADC) [7].

In relaxation based NMR approaches, the *T*_2_ [6] or *T*_1_ [8, 9] relaxation times of the extracellular water are selectively altered by a relaxation agent that cannot penetrate the cell membrane. The signal decay depends on the concentration of relaxation agent and on the exchange rate constant. The exchange can be estimated based on the two-compartment exchange model [8]. This approach is particularly suited to quantify relatively fast exchange, occurring on a 10 ms time-scale, like in the case of red blood cells [4, 5]. However, the invasive nature of this approach has been highlighted in a recent comparative study [10].

Diffusion NMR [11, 12] offers a non invasive alternative to measure exchange [13], relying on a naturally occurring difference in water ADC between the intra- and extracellular compartments. As measured by a pulsed gradient spin echo (PGSE) sequence, the intracellular water ADC, *D*_i_, is lower than that of the extracellular water, *D*_e_, due to restricted diffusion caused by the cell membranes [14]. The effects of exchange can be analysed using the Kärger model [13, 15]. Although the Kärger model [15] considers Gaussian diffusion in two exchanging compartments, it is used in several applications [7, 16–24]. The Kärger model can be modified to account for restricted diffusion [25] and for effects of different relaxation rate constants in the different compartments [26–28]. The rate of exchange is proportional to the membrane permeability, *P*. The average residence time for water molecules inside the cells, known as the intracellular lifetime, *τ*_i_, is inversely proportional to permeability, so that 1/*τ*_i_ = *PA*/*V*, where *A* is the membrane surface area and *V* is the intracellular volume [29, 30].

In contrast to the relaxation based approach, the diffusion exchange NMR is better suited for systems with relatively slow exchange, occurring on a time-scale of several 100 ms, which is inaccessible by the relaxation-based approach. In such conditions, a particularly advantageous method is the filter-exchange spectroscopy (FEXSY) introduced by Åslund *et al*. [19]. FEXSY is inspired by the diffusion-diffusion correlation experiment [17] using double diffusion encoding (DDE) [31] separated by a mixing time, *t*_m_. This allows to disentangle the effects of time-dependent diffusion and exchange. Compared to the full diffusion-diffusion correlation, FEXSY uses a much faster sparse sampling protocol [32], where the first diffusion encoding is applied as a low-pass diffusion filter that selectively attenuates the signal from compartments with high ADC. Furthermore, FEXSY analysis does not rely on inverse Laplace transform and thus avoids its inherent pitfalls [32].

FEXSY is suited to quantify intracellular lifetimes of approximately 200 to 2000 ms, such as for example in baker’s yeast cells [19–21] or in human breast cells [20, 22]. Due to it’s non-invasive nature, the FEXSY protocol can be adapted for constraints of human clinical and preclinical imaging systems [20, 33]. The imaging implementation of FEXSY, called filter exchange imaging (FEXI), can be applied *in vivo* to map apparent exchange rates in e.g. human brain [21, 24] or in human embryonic kidney cells [23].

The methodology proposed in this article was motivated by experiments designed to study water transport abilities of different types of human aquaporins. The results of this study will be presented elsewhere (Palmgren M, Hernebring M, Eriksson S, Elbing K, Geijer C, Lasič S, Dahl P, Hansen JS, Topgaard D, Lindkvist-Petersson K, unpublished work, 2017). Several model systems have been used to investigate the properties of water transport across the membranes of cells. In particular, *Xenopus laevis* oocytes and liposomes are systems commonly used to study the water transport activity of aquaporins [34, 35]. In such studies, water transport is commonly measured by detecting the swelling/shrinkage of the oocytes/liposomes upon an applied change in osmolality. In these experiments, water transport is quantified indirectly in terms of the osmotic water permeability coefficient. In contrast, the NMR methodology detects diffusional permeability coefficients. In our study, we use yeast cells (*Pichia pastoris*) as a model system. These cells can be modified using a methanol induced promoter to heterologously express human aquaporins [36, 37]. The advantage of using yeast cells compared to for instance red blood cells is the possibility to comparably easy remove all endogenous genes and insert genes of interest. In addition, yeast is a suitable system to over-express heterologous proteins at sufficient levels [37]. The intracellular lifetimes of these cells are expected to be at a 100 ms time-scale and extending beyond 1 s for cells without aquaporins, which is 10 to 100 times longer than in red blood cells. In such conditions, the FEXSY method offers an ideal opportunity to non-invasively study the transport mechanisms in equilibrium-exchange conditions.

In a multi-compartment system, the diffusion and relaxation effects can be different for each compartment. They can be quantitatively resolved only by encoding for both effects in a single two-dimensional *D* − *T*_2_ correlation experiment [32, 38–43]. In systems previously studied with FEXSY, the *T*_2_ relaxation was considered identical in all compartments [19]. However, different *T*_2_ relaxation for the intra- and extracellular water can cause bias when estimating the intracellular lifetime. In our preliminary experiments on genetically modified yeast cells, we observed large differences in the intra- and extracellular *T*_2_ values. For a quantitative comparative study, addressing the bias due to different relaxation times is crucial.

Here we introduce an augmented FEXSY protocol, which can be applied in comparative studies of aquaporins and cell cultures in general. To allow measurements of samples with short *T*_2_, we implemented the FEXSY sequence with a double pulsed gradient stimulated echo (double PGSTE) [44]. Most importantly, we present a method that can account for the confounding effects of different values of *T*_2_ and yield a bias-free estimate of the intracellular lifetime. The key to our novel method is a combination of the FEXSY and the PGSE based *D* − *T*_2_ correlation measurements. For our proof-of-concept, we used genetically modified *Pichia pastoris* yeast cells with deletion of the intrinsic aquaporins (*aqy1*Δ*agp1*Δ). For comparison, also cells with deletion of intrinsic and expression of human aquaporin (*aqy1*Δ*agp1*Δ + hAQP1) where used.

## Theory

**Fig 1:**
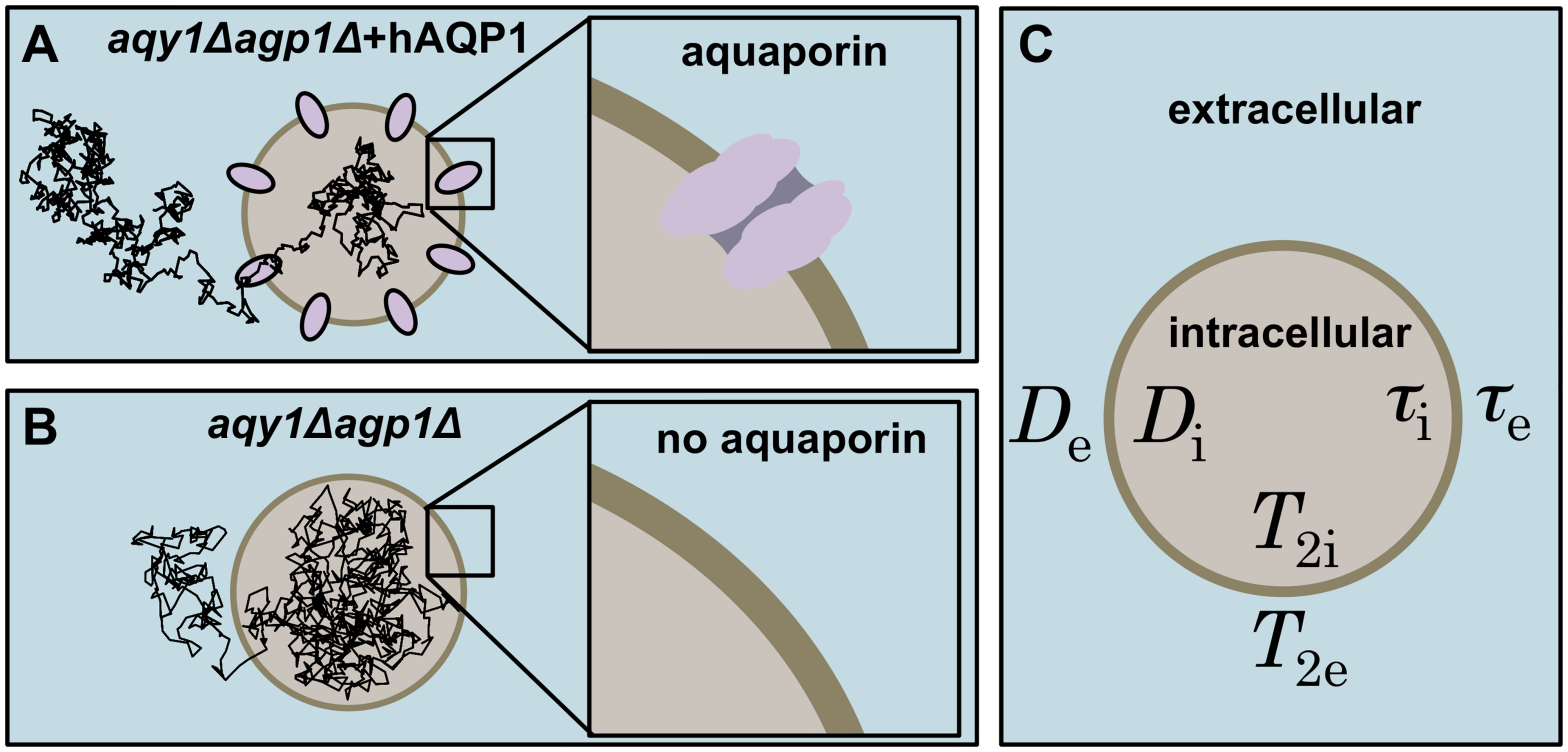
Illustration of the intracellular and extracellular compartments separated by cell membranes in a cell suspension. (A) *aqy1*Δ*agp1*Δ + hAQP1 are yeast cells with aquaporins and (B) *aqy1*Δ*agp1*Δ are yeast cells without aquaporins. Diffusion trajectory of one water molecule is shown inside the cells. (C) The different parameters in the intracellular/extracellular compartments are: apparent diffusion coefficients, *D*_i_/*D*_e_, transverse relaxation times, *T*_2i_/*T*_2e_, lifetimes of water molecules, *τ*_i_/*τ*_e_.

A schematic of the cell suspension system is shown in Fig 1. The system parameters for the intra- and extracellular compartments affecting the signal attenuation in the PGSE and FEXSY sequences are listed in Fig 1C. Cell membranes with and without aquaporins represent the *aqy1*Δ*agp1*Δ + hAQP1 and *aqy1*Δ*agp1*Δ yeast cells used in this article (see Fig 1A and 1B). The trajectories illustrate the different intracellular lifetimes for the two types of cells.

Our analysis uses an approximate and phenomenological approach to quantify the effects of diffusion, relaxation and exchange in a two-compartment system. We apply a similar mathematical formalism as in the stochastic treatment of relaxation in multiple phase systems [45] or in the analysis of multi-site chemical exchange based on the matrix formulation of the Bloch equations [46]. The time evolution of a spin-system, e.g. undergoing relaxation, is rigorously described in terms of the density operator subjected to the Liouville—von Neuman equation using evolution superoperators [47], often also called propagators [48]. In our analysis, we use operators in a more general mathematical sense, which here take the form of matrix exponentials. The signal evolution can thus be described as a composite of sequential operators, which include effects of diffusion, relaxation and exchange.

During an NMR pulse sequence, the magnetization experiences several coherence pathways, which are selected by phase cycling of the radio frequency pulses [11]. Rather than following the longitudinal and traverse components of different magnetization pathways, we consider the resulting signal magnitudes from intracellular and extracellular compartments, denoted with subscripts i and e, respectively. The acquired signal is given by contributions from the two compartments as *S* = **1** ⋅ **S**, where **S** = (*S*_i_, *S*_e_)^T^ and **1** = (1, 1). For convenience of notation, we use the transpose operator, denoted by the superscript *T*, to change row vectors into column vectors. With **S**_0_ = (*S*_0i_, *S*_0e_)^T^ we denote signal contributions that are not affected by diffusion, *T*_2_ relaxation or exchange. Assuming equal *T*_1_ for both contributions, the effects of *T*_1_ can be factored into **S**_0_. We further assume that the gradient pulses are short relative to the relaxation and exchange time-scales. This is a good approximation in our experimental conditions considering gradient pulses of 1.2 ms and it is used in the well-known and commonly applied Kärger model [13, 15].

**Fig 2:**
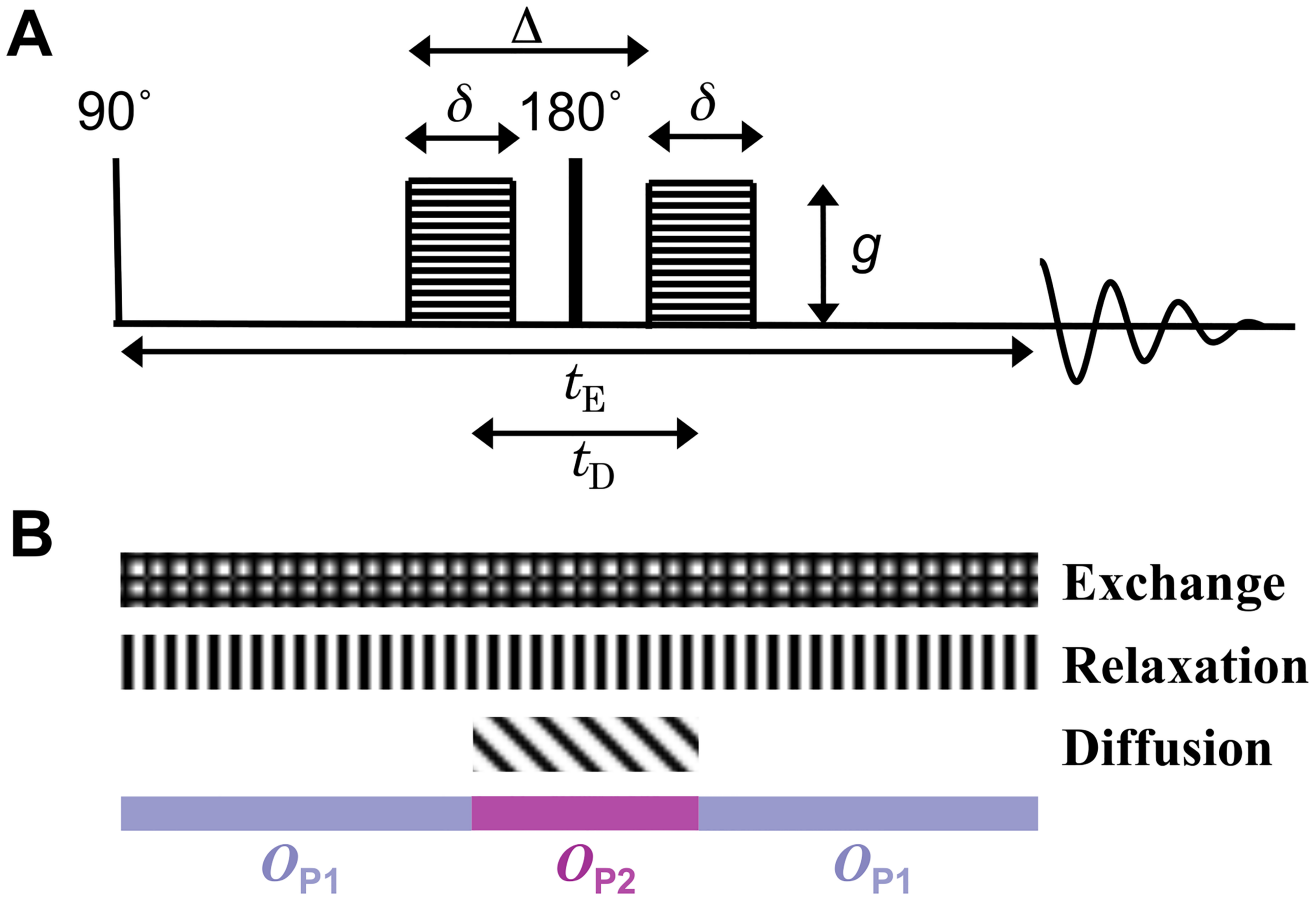
Schematic representation of the PGSE experiment. (A) The PGSE pulse sequence with gradient pulse duration *δ*, interval between the onset of the gradient pulses Δ, effective diffusion encoding time *t*_D_, gradient strength *g*, and echo time *t*_E_. (B) The different patterned blocks mark the time intervals when the signal is subjected to the evolution operators for exchange, *T*_2_-relaxation and diffusion. The coloured blocks show the time intervals during which the operators defined in Eqs 9 and 10 apply.

**Fig 3:**
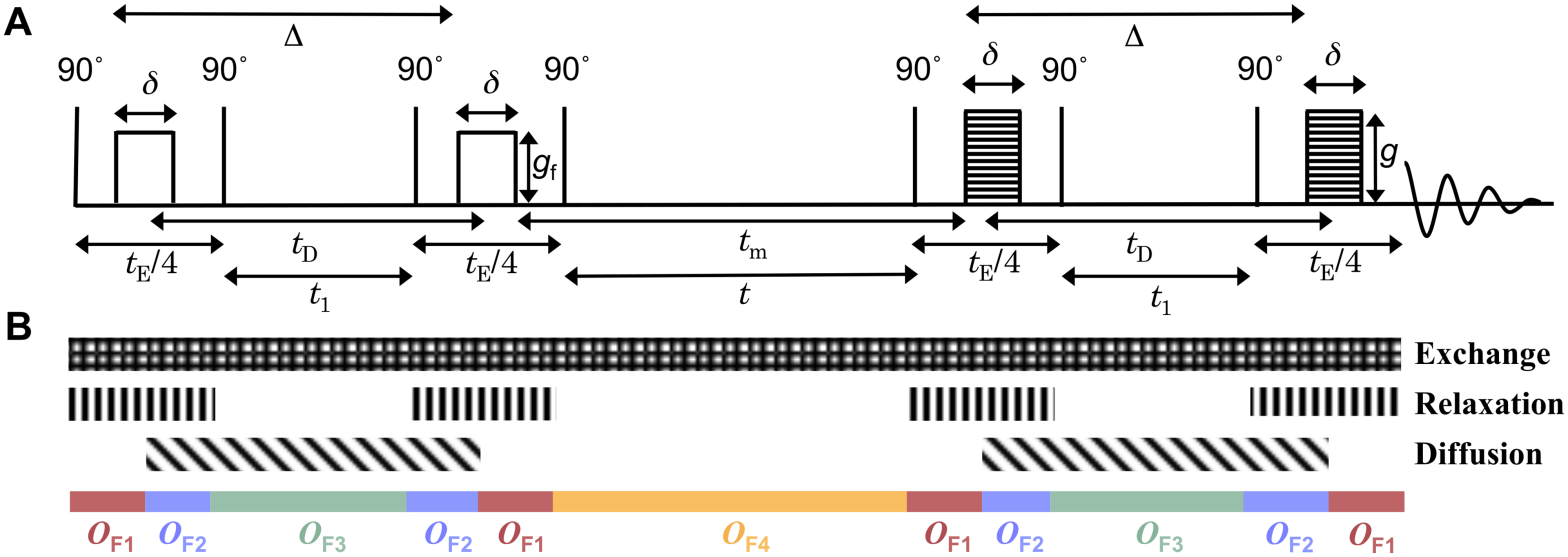
Schematic representation of the FEXSY experiment. (A) The FEXSY sequence with gradient pulse duration *δ*, interval between the onset of the gradient pulses Δ, effective diffusion encoding time *t*_D_, gradient strength of the filter block *g*_f_, gradient strength of the diffusion encoding block *g*, and the mixing time *t*_m_. Magnetization is affected by longitudinal relaxation during times *t* and *t*_1_ and by transverse relaxation during echo time, *t*_E_. (B) The different patterned blocks mark the time intervals when the signal is subjected to the evolution operators for exchange, *T*_2_-relaxation, and diffusion. The coloured blocks show the time intervals during which the operators defined in Eqs 12–15 apply.

The theoretical section is divided in three parts: (i) introducing operators to calculate the signal evolution due to diffusion, relaxation and exchange; (ii) applying the operators to predict signal attenuation in PGSE (Fig 2) and FEXSY (Fig 3) sequences, which is used in data fitting; (iii) obtaining analytic expressions to estimate the bias of the intracellular lifetime as a function of the difference in relaxation rate constants.

### Operators for diffusion, relaxation and exchange

#### Diffusion

To encode diffusion information, both the PGSE (Fig 2) and the FEXSY (Fig 3) sequences employ pulsed magnetic field gradients during the diffusion encoding blocks. The signal attenuation is controlled by the pulse duration, *δ*, separation in time, Δ, and amplitude, *g*, of the gradient pulses (see Figs 2 and 3). For low *g*, the signal attenuation is proportional to the diffusion weighting *b* = *q*^2^ *t*_D_, where *q* = *γgδ*, *γ* the gyromagnetic ratio and *t*_D_ = (Δ − *δ*/3) the effective diffusion time. Considering only diffusion, the signal evolution is given by

$$\begin{array}{c} S=e^{-q^{2}Dt_{D}}S_{0}, \end{array}$$ (1)

where

$$\begin{array}{c} D=\left(\begin{array}{cc} D_{i} & 0\\ 0 & D_{e} \end{array}\right). \end{array}$$ (2)

Note that the restricted diffusion effects are accounted for by the ADCs, *D*_i_ and *D*_e_, which depend on *δ* and *t*_D_ [49].

#### Relaxation

When the intracellular transverse relaxation times, *T*_2i_ and *T*_2e_ are different, the signal evolution during the transverse encoding echo time, *t*_E_, is given by

$$\begin{array}{c} S=e^{-Rt_{E}}S_{0}, \end{array}$$ (3)

where

$$\begin{array}{c} R=\left(\begin{array}{cc} R_{2i} & 0\\ 0 & R_{2e} \end{array}\right). \end{array}$$ (4)

Here *R*_2i,e_ = 1/*T*_2i,e_ are the relaxation rate constants for the respective compartments. The difference in relaxation rate constants is given by Δ*R*_2_ = *R*_2i_ − *R*_2e_.

#### Exchange

We describe the effects of exchange in a two-compartment system according to the first order kinetics [45, 50]. Due to exchange effects alone, occurring during a time interval *t*, the signal evolution is given by

$$\begin{array}{c} S=e^{-Kt}S_{0}, \end{array}$$ (5)

where

$$\begin{array}{c} K=\left[\begin{array}{cc} k_{\text{ie}} & -k_{\text{ei}}\\ -k_{\text{ie}} & k_{\text{ei}} \end{array}\right]. \end{array}$$ (6)

Here *k*_ie_ and *k*_ei_ are the intracellular to extracellular and the reverse exchange rate constants, respectively. The corresponding lifetimes are given by *τ*_i_ = 1/*k*_ie_ and *τ*_e_ = 1/*k*_ei_. The exchange matrix **K** fulfills the detailed equilibrium condition [51]

$$\begin{array}{c} {KS}_{0}=0. \end{array}$$ (7)

The intra- and extracellular signal fractions are given by *f*_i_ = *S*_i_/(*S*_e_ + *S*_i_) and *f*_e_ = 1 − *f*_i_. Considering the equilibrium condition in Eq 7 and the total exchange rate constant, *k* = *k*_ie_ + *k*_ei_, the exchange matrix can be expressed as

$$\begin{array}{c} K=k\left[\begin{array}{cc} f_{e}^{\text{eq}} & f_{e}^{\text{eq}}-1\\ -f_{e}^{\text{eq}} & 1-f_{e}^{\text{eq}} \end{array}\right], \end{array}$$ (8)

where $f_{e}^{\text{eq}}$ is the extracellular signal fraction at equilibrium.

### Signal evolution during PGSE and FEXSY

For the PGSE sequence, the signal evolution is given by the composite of operators *O*_**P1**_ and *O*_**P2**_ acting during three intervals: before(*O*_**P1**_), during(*O*_**P2**_) and after(*O*_**P1**_) the diffusion encoding (see Fig 2). We assume gradient pulses to be short, which is reflected in the instantaneous changes in the coloured blocks shown at the bottom of Fig 2 representing the three time intervals mentioned above. The *O*_**P1**_ operator includes the effects of relaxation and exchange,

$$\begin{array}{c} O_{P1}=e^{-\frac{1}{2}\left(R+K\right)\left(t_{E}-t_{D}\right)}. \end{array}$$ (9)

The operator *O*_**P2**_ includes the effects of relaxation, exchange and diffusion,

$$\begin{array}{c} O_{P2}=e^{-\left(R+K+q^{2}D\right)t_{D}}. \end{array}$$ (10)

The signal evolution in PGSE is given by

$$\begin{array}{c} S_{\text{PGSE}}=O_{P1}O_{P2}O_{P1}S_{0}. \end{array}$$ (11)

The PGSE sequence can be used to estimate Δ*R*_2_ based on the diffusion contrast between the two compartments. The diffusion and relaxation effects can be disentangled in a *D* − *T*_2_ correlation experiment [32, 43, 52, 53], where the diffusion weighting is kept constant, by using constant *δ* and Δ, while varying the relaxation weighting through *t*_E_.

For the FEXSY sequence, the signal evolution is given by the composite of operators *O*_**F1**_, *O*_**F2**_, *O*_**F3**_ and *O*_**F4**_. The time intervals during which these operators act and the effects that they include are depicted in Fig 3. Also in this case the assumption of short gradient pulses is reflected in the coloured blocks shown at the bottom of Fig 3. The operator *O*_**F1**_ includes the effects of relaxation and exchange,

$$\begin{array}{c} O_{F1}=e^{-\frac{1}{2}\left(R+K\right)\left(t_{E}/2+t_{1}-t_{D}\right)}, \end{array}$$ (12)

the operator *O*_**F2**_ includes the effects of relaxation, exchange and diffusion

$$\begin{array}{c} O_{F2}=e^{-\frac{1}{2}\left(R+K+q^{2}D\right)\left(t_{D}-t_{1}\right)}, \end{array}$$ (13)

the operator *O*_**F3**_ includes the effects of exchange and diffusion

$$\begin{array}{c} O_{F3}=e^{-\left(K+q^{2}D\right)t_{1}}, \end{array}$$ (14)

and the operator *O*_**F4**_ includes only the effects of exchange

$$\begin{array}{c} O_{F4}=e^{-Kt}. \end{array}$$ (15)

Note that FEXSY is specifically sensitive to exchange due to the isolated exchange effects in operator *O*_**F4**_. The signal evolution in FEXSY is given by

$$\begin{array}{c} S_{\text{FEXSY}}=O_{F1}O_{F2}O_{F3}O_{F2}O_{F1}O_{F4}O_{F1}O_{F2}O_{F3}O_{F2}O_{F1}S_{0}. \end{array}$$ (16)

### Bias of the intracellular lifetime in the presence of relaxation rate differences

Let’s examine the bias of the exchange rate constant and the intracellular lifetime estimated by the FEXSY sequence in the presence of the difference in relaxation rate constants Δ*R*_2_. To obtain tractable analytical expressions in this step of analysis, we ignore the effects of exchange during the diffusion encoding blocks, similar as in the standard FEXSY analysis by Åslund et al. [19]. Hence, the patterned block representing exchange in Fig 3B does not apply in this analytical derivation. The diffusion effects can be accounted for implicitly by considering the extracellular signal fraction at equilibrium, $f_{e}^{\text{eq}}$, and the fraction perturbed by the diffusion filter block, *f*_e0_ at *t*_m_ = 0. Note that diffusion encoding is necessary to experimentally determine the signal fractions.

The signal contributions are given by

$$\begin{array}{c} S_{r}=S_{0}e^{-Rt_{E}}e^{-Kt_{m}}e^{-Rt_{E}}{\left(1-f_{e0},f_{e0}\right)}^{T}, \end{array}$$ (17)

where *S*_0_ is the normalization factor. The relaxation-affected signal fraction is given by Eq 17 as

$$\begin{array}{c} f_{e,r}=S_{r}\left(2\right)/\left(S_{r}\left(1\right)+S_{r}\left(2\right)\right). \end{array}$$ (18)

**Fig 4:**
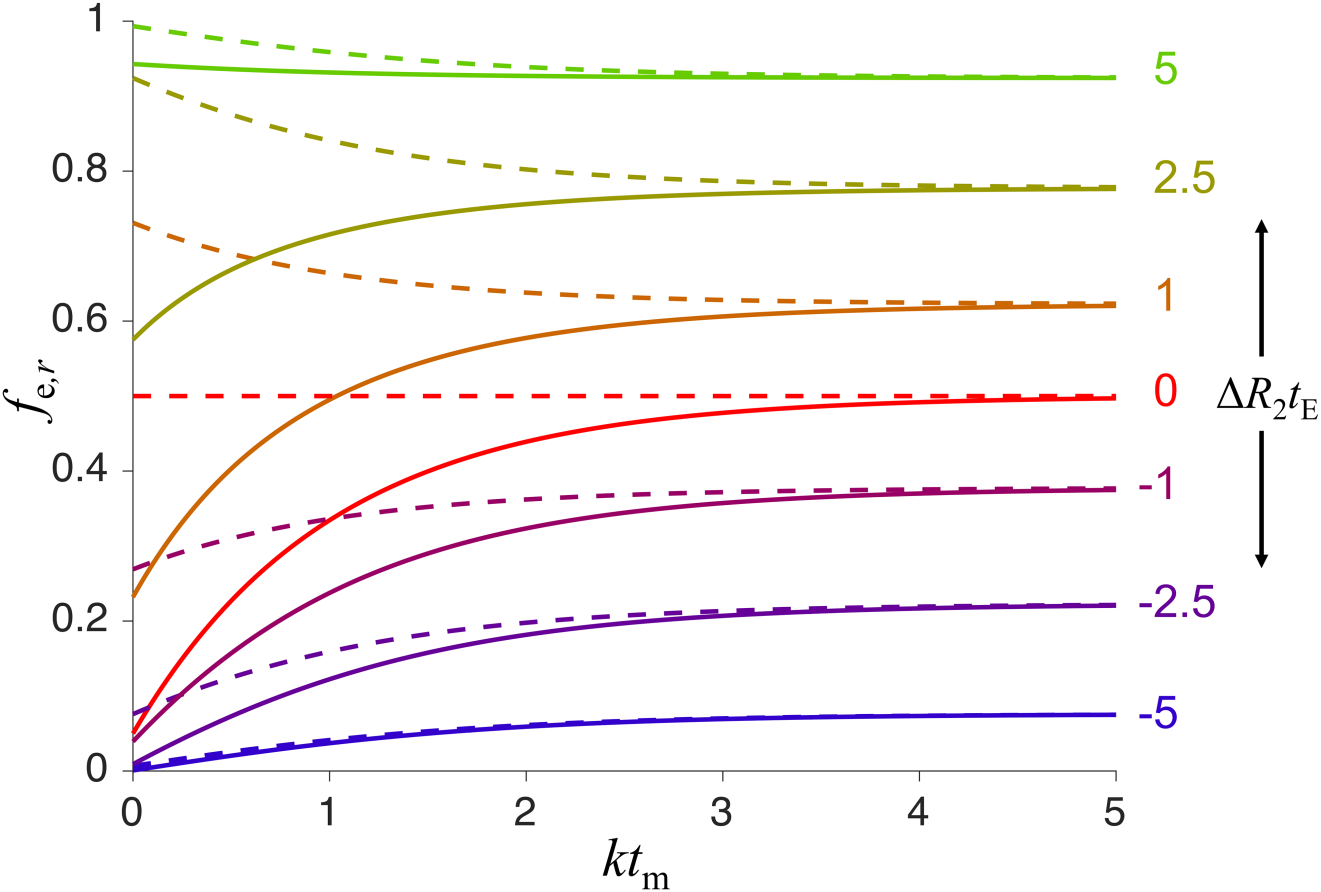
Relaxation-affected extracellular signal fraction as a function of *kt*_m_ for different values of Δ*R*_2_*t*_E_. Solid lines represent the case when diffusion filter gradients are applied in the first PGSTE block, while dashed lines are for the case without the filter gradients (see Fig 3). The results for different values of Δ*R*_2_*t*_E_ are labeled on the right ranging from −5 to +5 and color coded from blue to red for negative values and from red to green for positive values. In all cases, *f*_e0_ = 0.05 and $f_{e}^{\text{eq}}=0.5$.

Eqs 8, 17 and 18 yield the expression for the relaxation-affected extracellular fraction given by

$$\begin{array}{c} \begin{array}{cc} & f_{e,r}\left(f_{e}^{\text{eq}},f_{e0},kt_{m},r\right)=\\ & \left[\left(f_{e}^{\text{eq}}-f_{e}^{\text{eq}}e^{-kt_{m}}\right)\left(f_{e0}-1\right)-f_{e0}e^{r}\left(f_{e}^{\text{eq}}+e^{-kt_{m}}-f_{e}^{\text{eq}}e^{-kt_{m}}\right)\right]\\ & [\left(f_{e}^{\text{eq}}-f_{e}^{\text{eq}}e^{-kt_{m}}\right)\left(f_{e0}-1\right)-f_{e0}e^{r}\left(f_{e}^{\text{eq}}+e^{-kt_{m}}-f_{e}^{\text{eq}}e^{-kt_{m}}\right)\\ & +e^{r}\left(f_{e0}-1\right)\left(f_{e}^{\text{eq}}e^{-kt_{m}}-f_{e}^{\text{eq}}+1\right)+f_{e0}e^{-kt_{m}}\left(e^{kt_{m}}-1\right)\left(f_{e}^{\text{eq}}-1\right){]}^{-1}, \end{array} \end{array}$$ (19)

where *r* = Δ*R*_2_*t*_E_/2. The function in Eq 19 is shown graphically in Fig 4. For Δ*R*_2_ = 0, the asymptotic value of *f*_e_ at long *t*_m_ corresponds to the experimentally accessible value of *f*_e_ at short *t*_m_ and without the diffusion filtering [19]. This correspondence of *f*_e_ at short and long *t*_m_ no longer exists for |Δ*R*_2_| > 0 (compare solid and dashed lines in Fig 4).

In contrast to the case with Δ*R*_2_ = 0, when *f*_e_(*t*_m_) has an exponential dependence on *t*_m_ (see Eq 7 in [19]), the function *f*_e,*r*_(*t*_m_) does not have such a simple form. However, we can estimate the relaxation-affected exchange rate constant, *k*_r_, from the time dependence of *f*_e,*r*_ at short *t*_m_,

$$\begin{array}{c} k_{r}=-\lim_{t_{m}\to 0}\frac{\partial }{\partial t_{m}}\ln\left[\frac{f_{e,r}^{\text{eq}}-f_{e,r}\left(t_{m}\right)}{f_{e,r}^{\text{eq}}-f_{e0,r}}\right]. \end{array}$$ (20)

The expression 20 resembles the definition of the apparent exchange rate constant given by the time dependence of the ADC at short *t*_m_ (see Eq 11 in [20]). The $f_{e,r}^{\text{eq}}$ in Eq 20, determined as the asymptotic value of the relaxation-affected extracellular fraction at long *t*_m_, can be expressed with the relaxation non-affected value $f_{e}^{\text{eq}}$ as

$$\begin{array}{c} f_{e\infty ,r}^{\text{eq}}=\lim_{t_{m}\to \infty }f_{e,r}\left(t_{m}\right)=\frac{f_{e}^{\text{eq}}}{f_{e}^{\text{eq}}+\left(1-f_{e}^{\text{eq}}\right)e^{-r}}. \end{array}$$ (21)

When, on the other hand, $f_{e,r}^{\text{eq}}$ is determined at *t*_m_ = 0 and no diffusion filtering is applied in the first PGSTE block, as in our experiments, we have

$$\begin{array}{c} f_{e0,r}^{\text{eq}}=\lim_{t_{m}\to 0}f_{e,r}\left(t_{m}\right)=\frac{f_{e}^{\text{eq}}}{f_{e}^{\text{eq}}+\left(1-f_{e}^{\text{eq}}\right)e^{-2r}}. \end{array}$$ (22)

A similar expression is obtained for the relaxation-affected filtered fraction

$$\begin{array}{c} f_{e0,r}=\frac{f_{e0}}{f_{e0}+\left(1-f_{e0}\right)e^{-2r}}. \end{array}$$ (23)

It is interesting that the effect of relaxation difference, expressed by the factor exp(−2*r*) in Eqs 22 and 23, is reduced to the factor exp(−*r*) in Eq 21. This is a consequence of exchange between two compartments with different *R*_2_ occurring during long *t*_m_, which cancels the effect of Δ*R*_2_ during the first PGSTE block. Compare the dashed/solid lines at short/long *t*_m_ in Fig 4. Note that even though the effects of Δ*R*_2_ are different in the filtered and non-filtered experiments, the difference vanishes at long *kt*_m_.

To investigate the bias of *k*_*r*_ caused by Δ*R*_2_, we consider two cases. In the first case, we evaluate Eq 20 using the non-filtered signal fraction at long *t*_m_, $f_{e\infty ,r}^{\text{eq}}$ from Eq 21 and the filtered fraction *f*_e0,*r*_ from Eq 23 yielding

$$\begin{array}{c} k_{r}^{\infty }=k\frac{\left(f_{e0}e^{r}-f_{e0}+1\right)\left(f_{e}^{\text{eq}}e^{r}-f_{e}^{\text{eq}}+1\right)}{\left(f_{e0}e^{2r}-f_{e0}+1\right)}. \end{array}$$ (24)

In the second case, when the experimentally accessible $f_{e0,r}^{\text{eq}}$ from Eq 22 is considered, we get

$$\begin{array}{c} k_{r}^{0}=\frac{-ke^{r}\left(f_{e0}e^{r}-f_{e0}+1\right)\left(f_{e}^{\text{eq}}e^{r}-f_{e}^{\text{eq}}+1\right)\left(f_{e}^{\text{eq}}-f_{e0}f_{e}^{\text{eq}}-f_{e0}e^{r}+f_{e0}f_{e}^{\text{eq}}e^{r}\right)}{\left(f_{e0}-f_{e}^{\text{eq}}\right)\left(f_{e0}e^{r}-f_{e0}+1\right)}. \end{array}$$ (25)

**Fig 5:**
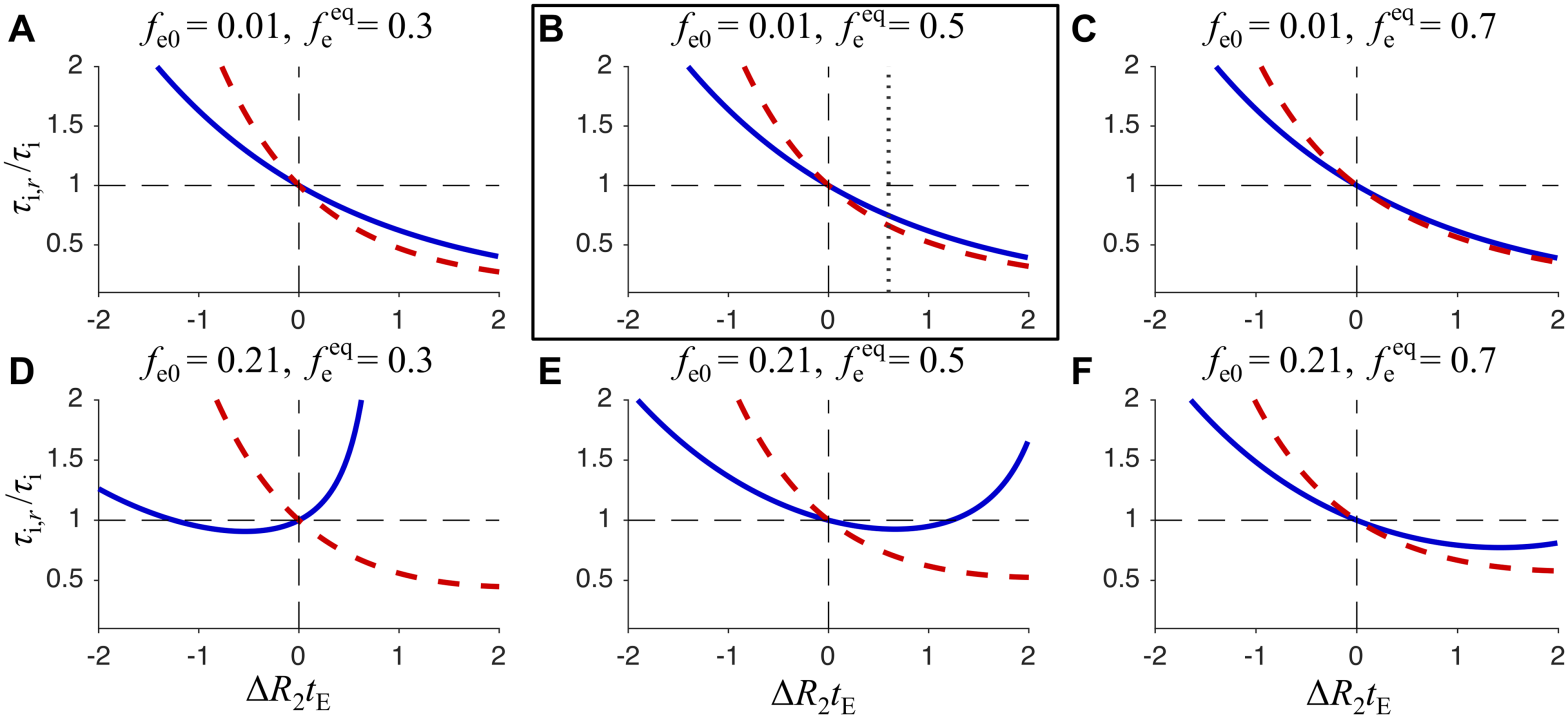
Relative bias of the intracellular lifetime as a function of Δ*R*_2_*t*_E_. The ratio of *τ*_i,*r*_/*τ*_i_ as a function of Δ*R*_2_*t*_E_, is shown for six different sets of *f*_e0_ and $f_{e}^{\text{eq}}$ values. The solid blue lines are calculated from Eq 25, while the dashed red lines are calculated from Eq 24. The framed plot shows the bias from low *f*_e0_ and 50% extracellular fraction, which approximately corresponds to our experimental conditions. The vertical dotted line is at Δ*R*_2_*t*_E_ = 0.6, which corresponds to echo time *t*_E_ = 17 ms and the difference in relaxation rate constants Δ*R*_2_ = 35 s^−1^. Here the relative bias (solid blue line) is about −20%.

From Eqs 24 and 25 we can calculate how the relaxation-affected intracellular lifetime, *τ*_i,*r*_ is related to the ground truth value *τ*_i_, i.e. the unbiased value that we wish to experimentally quantify. In Fig 5, the ratio of *τ*_i,*r*_/*τ*_i_ is shown as a function of Δ*R*_2_*t*_E_ for two different values of *f*_e0_ (rows) and three different values of $f_{e}^{\text{eq}}$ (columns). The red and blue curves correspond to Eqs 24 and 25, respectively.

It is clear from Fig 5 that the relative bias *τ*_i,*r*_/*τ*_i_ as a function of Δ*R*_2_*t*_E_ depends on *f*_e0_ and $f_{e}^{\text{eq}}$. This dependence is particularly pronounced when the equilibrium fraction is estimated at short mixing times (solid blue curves). The gray dotted line in the framed plot corresponds to our experimental value of Δ*R*_2_*t*_E_ = 0.6. When using the standard FEXSY analysis, ignoring the differences in relaxation rate constants and estimating the equilibrium fraction at short mixing times and for low *f*_e0_, the intracellular lifetime would in this case be underestimated by approximately 20%. However, for larger *f*_e0_ (see Fig 5E) the bias would only be −8%.

### Samples

Two different yeast cell strains *aqy1*Δ*agp1*Δ + hAQP1 and *aqy1*Δ*agp1*Δ were prepared by cultivating and inducing the yeast cells in methanol and harvesting after several hours. *Pichia pastoris* strains used in this study derive from the X33 strain (Invitrogen). The *aqy1*Δ*agp1*Δ + hAQP1 strain expresses human aquaporins while the *aqy1*Δ*agp1*Δ strain does not (see Fig 1).

The human aquaporins AQP1 with a His6 purification tag were cloned into pPICZB vector using restriction enzyme EcoRI and XbaI and transformed into *Pichia pastoris*. To identify clones that express the protein of interest, AQP transformants were spotted on YPD-agar plates supplemented with increasing zeocin concentrations (0, 0.5, 1.0 and 2.0 mg/ml). Cells were pre-grown in BMGY for 24 hours and protein production was induced with methanol to a final concentration of 0.25% (v/v) for 1 hour. The cells were harvested and washed with 20 mM HEPES pH 7.5, re-suspended in a 1:1 (v/v) solution with buffer, and transferred to 5 mm disposable NMR tubes. The tubes were centrifuged at 1000 *g* for 10 minutes to achieve a pellet with high concentration of cells (approximately 40% intracellular water). The excess fluid was removed from the tubes. The samples were kept on ice until measurement. Measurements were performed at 0 and 20°C.

### NMR experiment

The NMR experiments were performed on a Bruker 200 MHz Avance II system equipped with a DIF-25 probe capable of z-gradients up to 9.6 Tm^−1^. The temperature was controlled by a Bruker variable temperature unit with thermostatic air flow (±0.1°C). The samples were allowed to equilibrate inside the magnet 15 minutes before the experiments started.

The *D* − *T*_2_ correlation experiment (Fig 2) and the FEXSY experiment (Fig 3) were used for all samples. For the PGSE experiment, 10 different *t*_E_ values were used, logarithmically spaced from 5.5 to 82 ms. For each *t*_E_, the gradients were incremented logarithmically in 10 steps from 0.096 to 9.6 T m^−1^, yielding *b* values from 6.1 ⋅ 10^6^ to 2.2 ⋅ 10^10^ s m^−2^. The diffusion encoding timing parameters *δ* and Δ were kept constant during the experiment at 1.2 ms and 2.7 ms, respectively. The short Δ used here was necessary to achieve the shortest echo time, *t*_E_ = 5.5 ms, which allowed estimating short *T*_2_ values in the intracellular compartment.

For the FEXSY experiment, a double stimulated echo was used to minimize signal attenuation due to short *T*_2_. The sequence was performed with a 16-step phase cycle. The 8 steps of the cycle were used to eliminate artifacts that arise from unwanted coherence pathways as demonstrated by Khrapitchev and Callaghan [54]. The cycle was repeated 2 times with alternating direction of the magnetization z-storage by alternating the phase of the fourth 90° pulse. The signals from these two cycles were subtracted from each other to eliminate the contribution from the relaxed magnetization. The diffusion encoding time parameters were equal in both blocks and kept constant, *δ* = 1.2 ms and Δ = 8.4 ms. The strength of the filter gradient, *g*_f_, was set to 2.88 T m^−1^, resulting in *b*_f_ = 4.4 ⋅ 10^9^ s m^−2^. In the second diffusion encoding block the gradients were incremented logarithmically in 16 steps between 0.23 and 7.7 T m^−1^ yielding *b* values from 4.4 ⋅ 10^7^ to 4.9 ⋅ 10^10^ s m^−2^. The mixing times were 14, 54, 94 and 214 ms. For the non-filtered acquisition, used to estimate $f_{e0}^{\text{eq}}$, a filter gradient of 1% of *g*_f_ was used at *t*_m_ = 14 ms. The low filter gradient was used in addition to the 16 phase cycles to avoid contributions from unwanted magnetization pathways. An inversion recovery experiment with 16 recovery times between 10 and 2000 ms was performed to check that *T*_1_ was single-exponential.

### Data analysis

All data analysis was performed with Matlab (The MathWorks Inc., Natick, MA). Signal intensities were obtained by integrating over the water peak in the NMR spectra. A constrained global fit of Eqs 11 and 16 was made to the signal intensities from PGSE and FEXSY using a *lsqcurvefit* Matlab routine. Since the acquisition parameters were identical in both experiments, no signal weighting of the two datasets was necessary in the optimization routine. The common fit parameters, constrained to be identical for both datasets, were *τ*_i_, *τ*_e_, *T*_2i_ and *T*_2e_. Through the relationship shown in Eq 8 the value of $f_{e}^{\text{eq}}$ can be retrieved by $f_{e}^{\text{eq}}=\tau_{e}/\left(\tau_{i}+\tau_{e}\right)$. To account for different diffusion times in the two experiments $D_{i}^{\text{PGSE}}$, $D_{e}^{\text{PGSE}}$, $D_{i}^{\text{FEXSY}}$ and $D_{e}^{\text{FEXSY}}$ were used as free fit parameters. A single *S*_0_ value was used in PGSE, while *S*_0_(*t*_m_) was used in FEXSY to account for *T*_1_ relaxation. *S*_0_ here refers to the total initial signal, *S*_0_ = *S*_0i_ + *S*_0e_.

The errors in the fit parameters were estimated by least-square analysis of 1500 datasets obtained by bootstrap re-sampling [55] of the two experimental datasets, together consisting of 150 data points. To eliminate outlier fits, only the 85% of the fits with lowest *χ*^2^ were retained. From the retained fits, the means and standard deviations of the fit parameters were calculated. All the errors presented in this article refer to the standard deviation.

## Results and Discussion

**Fig 6:**
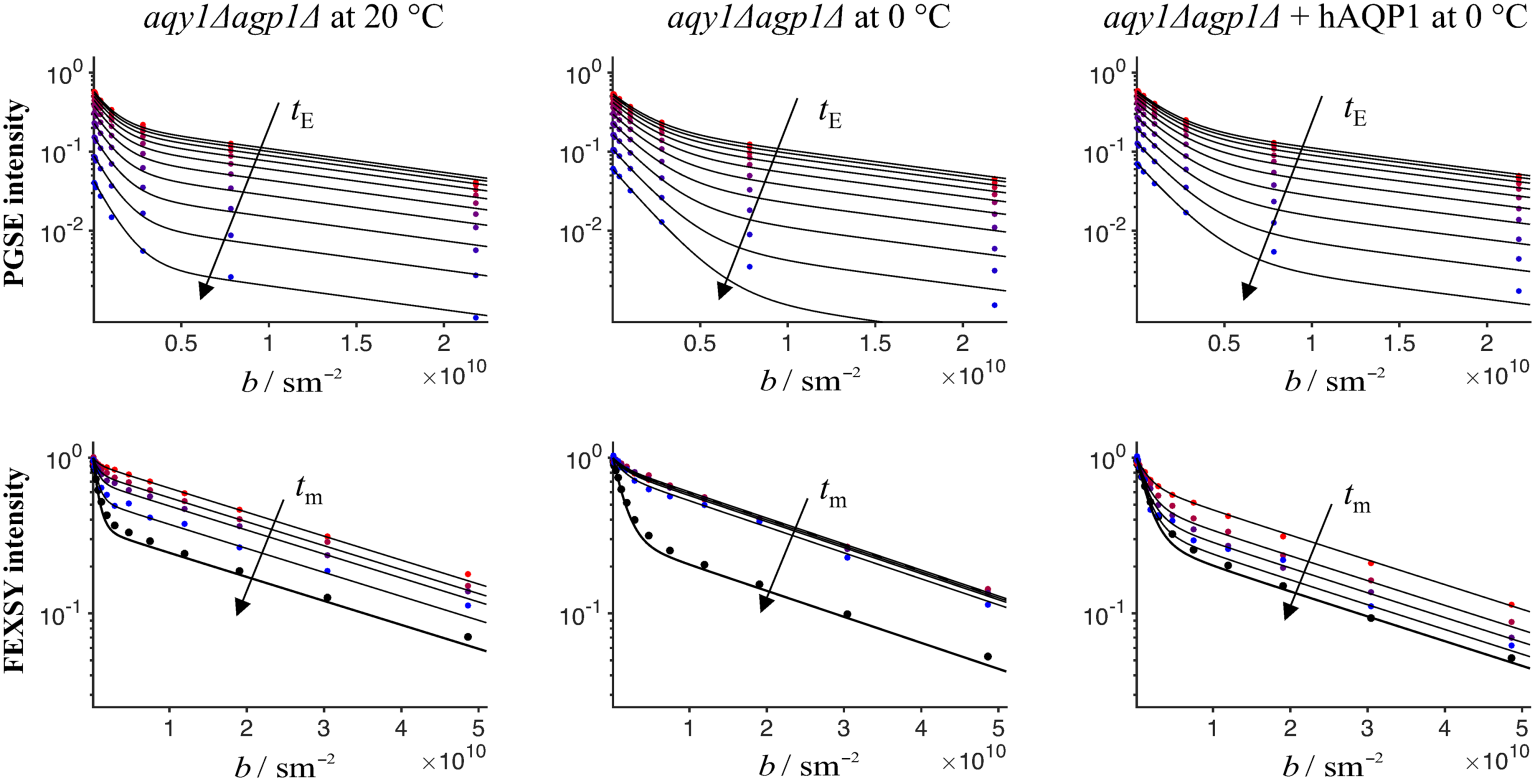
Signal vs. *b* from PGSE and FEXSY. Top row shows results of the PGSE experiment for increasing echo times, *t*_E_, indicated by the arrow. Bottom row shows results of the FEXSY experiment, where the arrow indicates the increasing mixing times, *t*_m_. Columns show results for different yeast strains at different temperatures. Columns 1 and 2: *aqy1*Δ*agp1*Δ measured at T = 20°C and at T = 0°C. Column 3: *aqy1*Δ*agp1*Δ + hAQP1 measured at T = 0°C. The result of a constrained global fit of Eqs 11 and 16 to the entire dataset (upper and lower plots) is shown by the solid black lines. For the PGSE experiment, all the signal intensities are normalized by the maximum *S*_0_ value at shortest *t*_E_, while for the FEXSY experiment, different normalization constants, *S*_0_(*b*_f_, *t*_m_), are used for different *b*_f_ and *t*_m_ values.

Due to the difference in relaxation rate constants, Δ*R*_2_, the standard FEXSY protocol yields biased estimates of *τ*_i_, as shown by the dashed red line in Fig 5. To avoid the bias, we introduced a complementary measurement of *D* − *T*_2_ correlation by a PGSE sequence with varying echo time. This measurement is specifically sensitive to *T*_2_ in the intracellular and extracellular compartments and can thus be used in combination with FEXSY to yield accurate quantification of *τ*_i_. The analysis is done by a global constrained fitting of Eqs 11 and 16 to the entire dataset consisting of PGSE and FEXSY data. The model accounts for both exchange and relaxation, *τ*_i,e_ and *T*_2i,e_, which are constrained to be identical for both experiments. The data and the fitting results for three experiments (*aqy1*Δ*agp1*Δ @ 0°C, *aqy1*Δ*agp1*Δ @ 20°C and *aqy1*Δ*agp1*Δ + hAQP1 @ 0°C) are shown in three columns of Fig 6. The upper row refers to the PGSE experiment and the lower row to the FEXSY experiment.

To maximize the contrast between *D*_i_ and *D*_e_ in FEXSY, longer *t*_D_ is favourable. On the other hand, a short *t*_D_ is favoured in the PGSE sequence to minimize the effect of exchange during the diffusion encoding and allow for the shortest possible *t*_E_ to be used. In our experiments, we empirically optimized the *t*_D_ in PGSE and FEXSY sequences separately. However, the use of different *t*_D_ values might be disadvantageous for the global fit analysis, since this yields different ADCs for the two experiments, not allowing constraining *D*_i_ and *D*_e_ to be identical for both datasets. Alternatively, a PGSTE sequence with fixed *t*_D_ and variable *t*_E_ could be used instead of the PGSE sequence to ensure identical *D*_i_ and *D*_e_ values.

To aid visualization in Fig 6, all the signal intensities for the PGSE experiment were normalized by the maximum *S*_0_ value at the shortest *t*_E_, while for the FEXSY experiment, different normalization constants, *S*_0_(*b*_f_, *t*_m_), were used for different *b*_f_ and *t*_m_ values. The effect of *T*_1_ relaxation was included in the fit parameters *S*_0_ for PGSE and *S*_0_(*b*_f_, *t*_m_) for FEXSY. Although a single value of *T*_1_ was not assumed in our analysis, complementary inversion recovery experiments showed monoexponential signal relaxation with *T*_1_ ≈ 400 ms for all studied samples (data not shown).

The bi-modal attenuations observed in the first row of Fig 6 are due to the diffusion contrast between the intra- and extracellular compartments. For the PGSE experiment, signal intensities decrease with increasing *t*_E_ due to *T*_2_ relaxation, with a more pronounced decrease for the intracellular (high *b* values) compartment than the extracellular compartment (low *b* values). This clearly demonstrates that the intracellular compartment has a shorter *T*_2_ than the extracellular compartment. Varying the diffusion encoding, *b*, allows identifying the relaxation weighted contributions from the two compartments, while varying *t*_E_ at constant diffusion weighting allows quantifying *T*_2_ values for each of the two compartments.

For the FEXSY experiment, different attenuation curves correspond to different mixing times. The data with black markers was measured without the diffusion filter and has the highest signal-to-noise ratio. The equilibrium extracellular signal fraction, $f_{e}^{\text{eq}}$, estimated from these data, as in the standard protocol [19], is biased due to the difference in relaxation rate constants. In fact, these data yield the relaxation-affected value $f_{e0,r}^{\text{eq}}$, given by Eq 22. After diffusion filtering at short *t*_m_, the extracellular signal is significantly more reduced than the intracellular signal and consequently the attenuation curve becomes almost monoexponential. As *t*_m_ increases, the extracellular signal fraction increases through exchange. However, for *aqy1*Δ*agp1*Δ measured at 0°C the exchange is so slow that there is hardly any recovery of the extracellular signal at longer *t*_m_. At 0°C, *aqy1*Δ*agp1*Δ + hAQP1 exhibit an exchange rate constant that is comparable to that in the *aqy1*Δ*agp1*Δ at 20°C. For *aqy1*Δ*agp1*Δ + hAQP1 at 20°C the exchange was too fast to be measured with FEXSY (data not shown).

The reduction of the exchange rate constant with temperature, observed in our experiments, is consistent with the results on baker’s yeast presented in Ref. [19]. There, the Arrhenius analysis of cell membrane permeability indicates that a significant change in the activation energy occurs at low temperatures. This could be caused by conformational changes in aquaporins or, more likely, due to a phase transition in the membrane lipids, consistent with the permeability decrease of lipid bilayer [56].

In our experiments, the temperatures where chosen so that the same set of *t*_m_ in FEXSY allowed a precise quantification of exchange for both *aqy1*Δ*agp1*Δ + hAQP1 and *aqy1*Δ*agp1*Δ yeast strains. The quantitative results for *τ*_i_, *τ*_e_, *T*_2i_ and *T*_2e_ are summarized in Table 1. In *aqy1*Δ*agp1*Δ, which have no aquaporins, the exchange occurs by diffusion through the lipid bilayer or possibly through other transport proteins that might be present in the membrane [57]. In this case, the exchange rate constant was reliably quantified at 20°C (see left column of Fig 6), but it was evidently very slow at 0°C (see middle column of Fig 6). Although the exchange rate constant could not be quantified in this case, we can conclude that the *τ*_i_ and *τ*_e_ were longer than 1 second. The aquaporin called AQP1 is known to be a very good water transporter [58], which is confirmed by our results shown in the right column of Fig 6. For the cells with aquaporin measured at 20°C, the exchange rate constant was out of range accessible by our method, which is approximately 0.5–20 *s*^−1^, corresponding to *τ*_i_ values approximately in the range of 50–2000 ms. This result clearly confirms a very pronounced difference in the exchange rate constants between *aqy1*Δ*agp1*Δ + hAQP1 and *aqy1*Δ*agp1*Δ. Further interpretation of results in terms of permeability per aquaporin and the discussion about aquaporin properties will be presented elsewhere.

**Table 1:** Estimated lifetimes and *T*_2_ values for the intra- and extracellular water in different yeast samples. The lifetimes, *τ*_i_ and *τ*_e_ and relaxation times, *T*_2i_ and *T*_2e_ resulting from a global fit of Eqs 11 and 16 to the PGSE and FEXSY datasets shown in Fig 6. Uncertainties correspond to one standard deviation determined by the bootstrapping error analysis.

	*aqy1*Δ*agp1*Δ *T* = 20°C	*aqy1*Δ*agp1*Δ *T* = 0°C	*aqy1*Δ*agp1*Δ + hAQP1 *T* = 0°C
*τ*_i_ (ms)	260 ± 30	> 1000	100 ± 20
*τ*_e_ (ms)	380 ± 60	> 1000	140 ± 30
$f_{e}^{\text{eq}}$	0.59 ± 0.04	0.6 ± 0.2	0.58 ± 0.07
*T*_2i_ (ms)	18.1 ± 0.7	16.1 ± 0.3	16.7 ± 0.4
*T*_2e_ (ms)	35 ± 1	43.8 ± 0.6	52 ± 3

The intra- and extracellular *T*_2_ values shown in Table 1 correspond to Δ*R*_2_ values of 22–35 s^−1^. The vertical dotted line in the framed plot of Fig 5 indicates the Δ*R*_2_*t*_E_ = 0.6, which corresponds to Δ*R*_2_ = 35 s^−1^ in our experiments with *t*_E_ = 17 ms. At this Δ*R*_2_, we estimate that the intracellular lifetime would be underestimated by about 20%, if determined from the standard FEXSY protocol ignoring differences in relaxation rate constants. From measurements on 24 cell suspension with different strains of yeast cells (data will be published separately), we see that Δ*R*_2_ values are not specific to a certain yeast strain. In addition, Δ*R*_2_ can vary for different cultivations of the same strain. We speculate that *T*_2_ values are very sensitive to sample preparation. It is clear from our study that an unbiased quantification of exchange requires to properly account for Δ*R*_2_. This is particularly important when comparing different cell cultures, e.g. when transport properties of different aquaporins are of interest.

Our simplified analysis includes two key assumptions: (1) exchange between compartments with restricted and hindered diffusion occurs on a time scale that is slow compared to the diffusion encoding times and (2) relaxation times are slow compared to the gradient pulse duration. The accuracy and applicable range of our analysis should in future studies be thoroughly investigated, e.g. by Monte Carlo simulations, accounting for the effects of restricted diffusion, relaxation and exchange.

## Conclusions

Diffusion NMR offers a non-invasive means to quantify molecular exchange in equilibrium-exchange conditions. In several biological applications of interest, where exchange takes place on a relatively slow time-scale of several 100 ms, the FEXSY method is particularly advantageous. An example of such an application involves studying the transport properties of different types of human aquaporins, which can be expressed in genetically modified yeast cells.

In our experiments on genetically modified yeast cells, different *T*_2_ values in the intra- and extracellular compartments were observed. In this case, using the standard FEXSY protocol would yield intracellular lifetimes that are underestimated by approximately 20%. To avoid this significant bias, we combine FEXSY, a method that is specifically sensitive to exchange, with a *D* − *T*_2_ correlation experiment, which is specifically sensitive to transverse relaxation and can detect subtle differences in *T*_2_ values between compartments with different apparent diffusivities. The effects of diffusion, exchange and relaxation can be accounted for in both experiments by using the signal evolution operators for the intra- and extracellular contributions. We analyse the data by global fitting, constraining the lifetimes, *τ*_i_ and *τ*_e_, as well as the relaxation times, *T*_2i_ and *T*_2e_, to be identical for both datasets. We demonstrate this novel method in our proof-of-concept experiments. The results of an extended study involving several types of aquaporins will be presented elsewhere.

When using FEXSY, accounting for differences between intra- and extracellular relaxation times is necessary whenever an unbiased quantification of exchange is required. This is particularly crucial in studies comparing different cell cultures, where it cannot be safely assumed that the values of *T*_2_ or the differences between *T*_2i_ and *T*_2e_ are the same across different cultivations.

## Abbreviations

*aqy1*Δ*agp1*Δ: Genetically modified *Pichia pastoris* yeast cells with deletion of the intrinsic aquaporins
*aqy1*Δ*agp1*Δ + hAQP1: Genetically modified *Pichia pastoris* yeast cells with deletion of the intrinsic aquaporins and expression of human aquaporin
NMR: Nuclear magnetic resonance
PGSE: Pulsed gradient spin-echo
PGSTE: Pulsed gradient stimulated echo
ADC: Apparent diffusion coefficient
DDE: Double diffusion encoding
FEXSY: Filter exchange spectroscopy
FEXI: Filter exchange imaging
